# Towards Resolving the Complete Fern Tree of Life

**DOI:** 10.1371/journal.pone.0024851

**Published:** 2011-10-13

**Authors:** Samuli Lehtonen

**Affiliations:** Department of Biology, University of Turku, Turku, Finland; Biodiversity Insitute of Ontario - University of Guelph, Canada

## Abstract

In the past two decades, molecular systematic studies have revolutionized our understanding of the evolutionary history of ferns. The availability of large molecular data sets together with efficient computer algorithms, now enables us to reconstruct evolutionary histories with previously unseen completeness. Here, the most comprehensive fern phylogeny to date, representing over one-fifth of the extant global fern diversity, is inferred based on four plastid genes. Parsimony and maximum-likelihood analyses provided a mostly congruent results and in general supported the prevailing view on the higher-level fern systematics. At a deep phylogenetic level, the position of horsetails depended on the optimality criteria chosen, with horsetails positioned as the sister group either of Marattiopsida-Polypodiopsida clade or of the Polypodiopsida. The analyses demonstrate the power of using a ‘supermatrix’ approach to resolve large-scale phylogenies and reveal questionable taxonomies. These results provide a valuable background for future research on fern systematics, ecology, biogeography and other evolutionary studies.

## Introduction

Ferns (monilophytes *sensu* Pryer et al. [1]) comprise ca. 12,000 extant species [2] and are the closest living relatives of the seed plants [1]. The first molecular systematic studies on ferns were published in the mid 1990s [3]–[5], and set the direction for modern fern systematics. Since then, numerous molecular phylogenetic studies have either focused on certain classically defined fern groups by sampling members from the group studied, or tested the backbone fern classification by sampling exemplar species of higher taxa. Both kinds of studies have, however, specific limitations to recover the complete fern tree of life. Well-sampled analyses are crucial for understanding the lower level phylogenetic patterns, but due to their generally limited scope the higher level relationships remain untested. Conversely, the relationships between higher taxonomic ranks (such as genera or families) may be seriously obscured if only one or few representatives of each group are sampled [6], [7].

Both densely sampled yet taxonomically limited and phylogenetically broader studies of selected exemplar taxa have greatly improved our understanding of the evolutionary history of ferns and provided a backbone for their modern classification [8]. However, a different analytical approach is emerging. The so-called ‘supermatrix’ or ‘mega-phylogeny’ analyses, based on enormous sets of data, have been introduced as an approach to solve the major branches of, or even the complete, tree of life [9]–[17]. These studies have not only shown that phylogenetic analyses of massive data sets can be conducted in a reasonable amount of time, but they have also revealed the importance of adequate taxon sampling to resolve difficult phylogenetic questions. For example, Smith et al. [16] were able to reconstruct the phylogeny of major vascular plant lineages using the *rbcL* gene in a supermatrix analysis, whereas previous studies analyzing considerably fewer taxa required many more genes to reveal the same relationships. Despite the great advances in pteridology, the fern phylogeny with the highest number of taxa published so far [18] was based on no more than three genes and 400 species, representing only approximately 3% of the global fern diversity. Some large-scale supermatrix analyses have included more fern taxa, but were based on fewer genes [15], [16]. The number of publicly available sequence data is rapidly growing, and GenBank currently covers over one-fifth of the estimated global fern species diversity. The present study is aimed at inferring the first supermatrix-based fern phylogeny. The resulting phylogeny should help the identification of poorly sampled or resolved branches of the tree, as well as the definition of natural ingroups and the selection of appropriate outgroups for more detailed phylogenetic analyses. It is well known that some erroneous data will always enter into large databases, such as GenBank. The analysis of large datasets could in this sense also help to identify such problematic data. Furthermore, a supermatrix phylogeny should provide a valuable backbone for other evolutionary research, such as biogeographical, ecological, and community-level phylogenetic studies.

## Results

**Figure 1:**
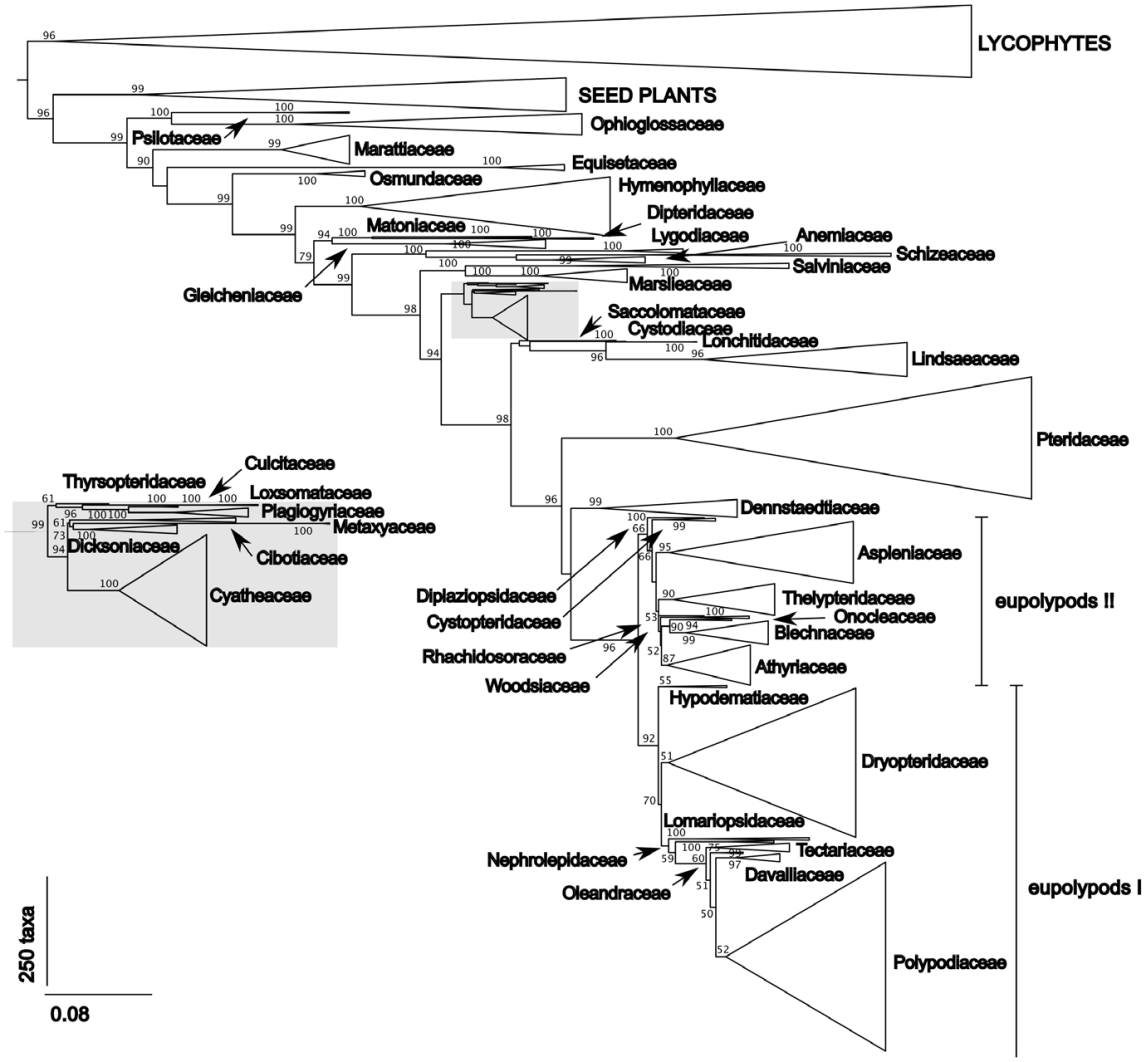
The ML tree showing the currently recognised fern families. Bootstrap support values greater than 50 are shown at nodes.

The combined four-gene (*atpA*, *atpB*, *rbcL*, *rps4*) data set included a total of 5,166 sequences (Dataset S1), hence the matrix of all 2,957 taxa by four genes had 6662 missing gene sequence entries (c. 56% missing data). Most taxa (91%) were represented by the *rbcL* gene, but the least sampled gene (*atpA*) was available for only approximately 18% of the taxa. Less than 10% of the sampled taxa were represented by all four genes, and 54% were represented only by a single gene. In most of the fern families at least some taxa were sampled for all markers, with two small families (Diplaziopsidaceae and Rhachidosoraceae) represented by the *rbcL* gene only and four other families (Psilotaceae, Schizeaceae, Cystodiaceae and Lomariopsidaceae) lacking one of the studied genes. The parsimony analysis of these data retained 124 equally parsimonious trees of 74,910 steps (Dataset S2). The final ML optimization likelihood score was −391724.512141 (Figure 1, Dataset S3).

Parsimony and ML trees were largely consistent with each other and with the prevailing view of the fern familial relationships [1], [18]–[20]. The parsimony analysis positioned horsetails (Equisetopsida) as a sister group to the Marattiopsida-Polypodiopsida clade, whereas ML placed them as sister to Polypodiopsida. These controversial groupings received low support values. Within the tree fern clade, ML and parsimony largely disagreed at the family level. Metaxyaceae was positioned as a sister to other tree ferns in the parsimony analysis, whereas in the ML tree the family was placed as sister to Dicksoniaceae. The clade composed of Thyrsopteridaceae and associated families in the ML tree also included Cibotiaceae and Dicksoniaceae in the parsimony analysis. Similarly, the two methods disagreed in the exact phylogenetic position of Dennstaedtiaceae and many small families, including Saccolomataceae, Cystodiaceae, Hypodematiaceae, Cystopteridaceae and Woodsiaceae. However, most of the incongruent groupings received less than 50% bootstrap support in both analyses, consistently with the observation that their relationships were also uncertain in previous studies [18], [23].

A recently published linear fern classification [8] was largely supported at the family level. At the generic level, however, improved sampling revealed several patterns that were inconsistent with previously published results and current fern taxonomy. Some of the most relevant results are shortly described here, otherwise readers are directed to trees available as supplementary information (Dataset S2, S3) and at TreeBase (http://purl.org/phylo/treebase/phylows/study/TB2:S11686). In Ophioglossaceae, the results contradicted those published by Hauk et al. [21] notably regarding the position of *Cheiroglossa*, which is here nested within *Ophioglossum*. In addition, *O. lusitanicum* L. was here grouped together with *Helmintostachys zeylanica* (L.) Hook. In the present study, the genus *Odontosoria* (Lindsaeaceae) was polyphyletic, and *Sphenomeris* was grouped with the *Tapeinidium*-*Osmolindsaea*-*Nesolindsaea* clade, thus contradicting the results of a recent study on Lindsaeaceae phylogenetics [22].

In Pteridaceae, all subfamilies accepted by Christenhusz et al. [8] were found to be monophyletic, although the monophyly of Cheilanthoidea had poor support. By contrast, numerous pteridoid genera, including *Adiantum*, were not monophyletic. The need for a generic redefinition within pteridoids has already been well recognized by earlier studies [8], [18], [23]–[27]. The relationship between pteridoids and dennstaedtioids was still ambiguous to date [23], and the present study also did not provide conclusive results. Pteridaceae was positioned as a sister group to eupolypods by parsimony, whereas ML supported Dennstaedtiaceae as sister to eupolypods. Both hypotheses received less than 50% support. The genus *Dennstaedtia* was paraphyletic as in previous studies [18], [28], due to the inclusion of the monophyletic *Microlepia*.

Eupolypods were separated into two clades, corresponding with eupolypods I and II [23]. Diplaziopsidaceae was resolved as sister to eupolypods II. Christenhusz et al. [8] included *Diplaziopsis*, *Homalosorus* and *Hemidictyum* within the Diplaziopsidaceae, but here *Hemidictyum* was supported as sister to Aspleniaceae as in Schuettpelz & Pryer [18] and in Kuo et al. [20]. *Hemidictyum* is therefore considered here as a member of Aspleniaceae and only *Diplaziopsis* and *Homalosorus* are within Diplaziopsidaceae, as in a recent analysis of the *matK* gene [20]. Family-level relationships mostly remained poorly supported or unresolved within both of the two large eupolypod clades.

Aspleniaceae was divided into three well-supported lineages, corresponding to *Hemidictyum* and two broadly-defined genera: *Hymenasplenium* and *Asplenium* [8], [18]. Several well-supported clades were also present within Thelypteridaceae, although not exactly matching the current generic classification. Similarly, previous studies have suggested that the current classification of Blechnaceae is unnatural [8]. In this study, the family was divided into three well-supported clades that did not correspond to the currently accepted generic limits (*Woodwardia*; *Salpichlaena*-*Stenochlaena*-*Blechnum* p.p.; *Blechnum* p.p.-*Brainea*-*Sadleria*-*Pteridoblechnum*). Within Athyriaceae, *Diplazium* was strongly supported as monophyletic, but *Cornopteris* was nested within *Athyrium* with a high level of support.

The two subfamilies of Dryopteridaceae [8] were monophyletic (with the exception of *Dryopteris inaequalis* (Schlecht.) Kuntze, which was placed in Elaphoglossoideae) in the ML analysis, albeit with a very poor support. In the parsimony analysis, on the other hand, the subfamily Elaphoglossoideae was divided into two groups with unresolved relationships with Dryopteridoideae. The subfamily Elaphoglossoideae included *Pleocnemia winitii* Holttum, the only member of its genus included in this study. Previous studies have considered *Pleocnemia* as a member of Tectariaceae [8], [23], [30]. At a generic level, *Polystichum* included *Cyrtomium* and *Cyrtogonellum*, *Arachnioides* included *Leptorumohra* and *Lithostegia*, and *Acrorumohra* was nested in *Dryopteris* (excluding *D. inaequalis*).

*Arthropteris* and *Psammiosorus* were mixed, but together formed a well-supported sister lineage to all other Tectariaceae. The proposed subfamilies of Polypodiaceae [8] were monophyletic in the ML analysis, except that *Synammia* was resolved as sister to Drynarioideae rather than being a member of Polypodioideae. The parsimony analysis did not support monophyletic Polypodiaceae, resulting in a largely unresolved topology within the eupolypods I. The current generic classification failed to delimit natural groups within Drynarioideae, Microsoroideae and Polypodioideae. The subfamily Loxogrammoideae was resolved as sister group to the remaining Polypodiaceae in the ML analysis.

## Discussion

The trees obtained here were generally consistent with the prevailing view of the molecular phylogeny of ferns [1], [18]–[20]. The taxonomic sampling employed here was almost seven-times broader than in the previous best-sampled fern phylogenetic analysis, hence providing a broader picture of fern phylogenetics, and enabling the investigation of the monophyly of currently accepted genera and families. However, despite the broad sampling, numerous fern groups remained poorly sampled and some phylogenetic relationships could not be completely resolved. For example, the families belonging to the eupolypods II group are well supported as monophyletic entities, but the relationships between them remained poorly established. The relationships among some of the early diverging polypods (Saccolomataceae, Cystodiaceae) were not unambiguously resolved and questions about a pteridoid-dennstaedtioid relationship still remained unanswered. Similarly to previous studies [1], [20], [27]–[30], the phylogenetic position of horsetails (Equisetaceae) remained controversial.

The observed uncertainty might, to some extent, reflect the large number of missing entries in the supermatrix. More than half of the taxa were represented by a single gene, *rbcL* being clearly the best-sampled marker. Those markers not as thoroughly sampled were, however, sampled rather evenly across the different fern lineages, so that very few families completely lacked data of one or more genes. Furthermore, Smith et al. [16] were able to resolve several difficult problems in the phylogeny of green plants by sampling the *rbcL* gene only, and it has been shown that supermatrix approach can handle even 90% missing data without loss of accuracy if the data available contain enough informative characters [11], [17], [32]–[35].

Most of the incongruent or poorly supported nodes in the present study connect very short internal nodes (as in eupolypods II), or very long terminal branches (e.g. *Equisetum*, Saccolomataceae), representing challenging situations for phylogenetic inference [36]–[38]. Therefore, it seems that the observed phylogenetic instability is not a result of the supermatrix approach *per se*, but more likely reflects a lack of suitable data in general. Poor support may, however, be linked to the supermatrix approach. Firstly, the large amount of missing data, which is a typical feature of supermatrices, automatically reduces re-sampling support [11]. Furthermore, in large data sets support values are generally expected to decline, partly because monophyly can be more easily rejected with increased taxon sampling [11], [39]. In addition, large data sets still provide serious computational challenges in multiple sequence alignment and tree search, and the necessary analytical short cuts may compromise some approaches and results [11], [12], [15]–[17]. To minimize alignment problems, only the best sampled protein coding genes were used here, and inserted gaps were treated as missing data. The method used to compile the supermatrix did compromise some of the study goals. First, the inclusion of all available sequence entries instead of only one per taxon would have been better to detect erroneous or misidentified sequences. This, however, would have greatly increased computational load, and shifted the main focus of the study from the phylogenetics to specimen identification. Another possible source of error may have resulted from the data concatenation: it was not verified whether the sampled genes were sequenced from the same voucher. Indeed, in many cases, data originating from different studies conducted by different research groups were combined. This may have resulted in error if different classifications were used in the original studies, or if identifications were not correct. In numerous cases taxa were listed in GenBank under various names, due for example to spelling errors or the use of different classifications. Whenever noticed, redundant names were eliminated, but solving this problem would require the use of taxon identifiers by GanBank enabling the automatic recognition of synonym names. Major concerns related to the present approach also include the exclusion of extinct fern lineages [10], [12], [31] and the complete reliance on plastid DNA data. A better understanding of the fern tree of life may provide a stronger background for comparative morphological analyses, hence enabling a more rigorous use of fossil and other morphological evidence in future studies. It would also be critical to test the current plastid-based fern phylogeny with one based on nuclear sequence data.

The advances in fern systematics over the past decades have provided a rather good taxonomic understanding at the family level, and the recently proposed fern classification [8] was largely supported by the current study. Generic delimitation, however, has remained ambiguous in a number of fern families [8], [23]. The analyses presented here shed new light on several unresolved issues, and can be used as a starting point to a more robust classification at this taxonomic level. A good example was that of Blechnaceae, a family composed of three well supported clades that (apart from *Woodwardia*) do not correspond well with the currently accepted generic classification.

Until recently, most of the molecular systematic studies of ferns were based on classical fern taxonomy. The most convenient way of overcoming the impact of outdated taxonomies, as well as detecting contaminated or misidentified sequences [11], [40], [41], is through the use of supermatrix analysis of all available data. The results presented here corroborated most recent findings in molecular fern systematics, but also provided a much wider view for future studies in fern evolution, taxonomy, and beyond. Instead of relying on the classical fern taxonomy, pteridologists can now select proper outgroups and delimit their ingroups in an appropriate way from an evolutionarily perspective. As yet, only about one-fifth of the extant fern diversity is currently covered by GenBank, but the road is open for a fully sampled fern tree of life, and ultimately, for a natural fern classification.

## Materials and Methods

Sequence data was retrieved from GenBank release 176 (Feb. 23, 2010) using PhyLoTA browser (http://phylota.net). PhyLoTA assembles BLAST clustering for all sequences in the GenBank release file [42]. Clusters corresponding to four protein coding plastid genes, *rbcL*, *rps4*, *atpA*, and *atpB*, were downloaded for root node “Moniliformopses”. This data set was further supplemented by downloading *rbcL* data of Japanese ferns [43] and adding several fern sequences produced with standard methods and primers [3], [19], [44]–[49] in our laboratory and submitted to GenBank, but not yet available on the queried release (GenBank accession numbers HQ157300–HQ157307, HQ157324–HQ157330, HQ157332–HQ157334, HQ245099–HQ245103, HQ680978). When multiple sequences were available for one taxon, the most complete one was retained and the other sequences excluded. A few sequences in the preliminary test analyses were positioned into highly questionable taxonomic groups, and these apparently misidentified or contaminated sequences were also excluded from the final analyses. The finally accepted fern sequences (2,656 taxa) were further supplemented with 301 outgroup taxa representing lycophytes (205 taxa), angiosperms (61 taxa) and gymnosperms (35 taxa).

Multiple sequence alignments were produced for each data set with Muscle [50] using default settings followed by one round of refinement. Due to variable sequence completeness all the alignments had high amounts of missing data at the 5′ and 3′ ends. These ambiguous regions were eliminated from the final data sets after visual inspection, as well as ambiguously aligned segment within the *rps4* gene. However, possible errors in the sequences (such as stop-codons) were not investigated. Indels inserted during the sequence alignment were treated as missing data in the corresponding phylogenetic analyses. Because all the markers included were plastid genes they were expected to share a common evolutionary history and were analyzed simultaneously. Aligned sequence matrices were concatenated with SequenceMatrix software [51]. In total, the data set consisted of 2,957 taxa (*rbcL* 2,681; *rps4* 1,134; *atpB* 825; *atpA* 526 taxa) and 4,406 aligned base pairs of molecular data (*rbcL* 1,332; *rps4* 379; *atpB* 1,188; *atpA* 1,507 bp). The aligned data matrices and resulting trees are available at TreeBASE (http://purl.org/phylo/treebase/phylows/study/TB2:S11686?x-access-code=133464583a4ffd664e66526ec5a0f6f5&format=html, [52]).

Phylogenetic analyses were performed for the concatenated supermatrix under equally weighted parsimony criteria using TNT [53] and maximum likelihood criteria using RAxML [54]. In the parsimony analyses 500 ‘new technology’ [55], [56] search replications were used as a starting point for each hit. These replications saved no more than 10 trees per replication, and were run until the best score was hit 10 times, using TBR-swapping, random and constraint sectorial searches, five ratchet iterations, and five rounds of tree fusing (*xmult = repl 500 hits 10 css rss ratchet 5 fuse 5 hold 10*). The memory was set to hold 80,000 trees. Branch support was evaluated by running 500 bootstrap replicates. TBR-swapping, sectorial search, and five rounds of tree fusing were employed in each replicate (*resample = boot replications 500 savetrees [xmult = rss css fuse 5]*). Maximum likelihood (ML) analyses were performed using the parallel Pthreads-version of the computer program RAxML 7.2.8 [54], [57] running in 2×2.26 GHz Quad-Core Intel Xeon Macintosh with 8 GB of RAM. The search was initiated with 500 rapid bootstrap replications followed by a thorough ML search on the original alignment (*-T 16 -f a -x 12345 -p 12345 -# 500 -m GTRGAMMA*). Free model parameters were estimated by RAxML under the GTR+Γ model. This is the most commonly used model for real data sets, and provides good performance for large data sets [58].

Congruence among the data sets was examined by running parsimony bootstrap analyses for each gene separately [59]. Visual inspection of the family-level nodes did not reveal well-supported (>70% support) conflict at this phylogenetic level, with the exception of nested position of Lonchitidaceae within Lindsaeaceae in the *atpA* analysis (data not shown). At lower phylogenetic levels the highly variable taxon sampling made the assessment of phylogenetic conflict highly problematic, and simultaneous analysis of all data sets was considered appropriate based on family-level congruence.

## Supporting Information

Dataset S1 Concatenated supermatrix in Nexus-format (file can be opened after unzipping for example with Mesquite [60]).
(NXS)

Dataset S2 The strict consensus tree of parsimony analysis with bootstrap support values in Nexus-format (file can be opened for example with FigTree [61]).
(NXS)

Dataset S3 The ML tree with bootstrap support values in Nexus-format (file can be opened for example with FigTree [61]).
(NXS)
